# Multidimensional Measurements of Dysarthria in Myotonic Dystrophy Type 1

**DOI:** 10.1111/1460-6984.70239

**Published:** 2026-04-08

**Authors:** Sanne van Hellemond, Nicole Voet, Rosemarie Kroon, Yelda Sahin, Ilse Karnebeek, Marthè Nijkamp, Simone Knuijt

**Affiliations:** ^1^ MA Speech‐Language Pathology Radboud University Nijmegen Nijmegen the Netherlands; ^2^ Department of Rehabilitation, Donders Institute for Brain, Cognition and Behaviour Radboud University Medical Center Nijmegen the Netherlands; ^3^ Rehabilitation center Klimmendaal Arnhem the Netherlands; ^4^ Department of Neurology Radboud University Medical Center Nijmegen the Netherlands; ^5^ Stem‐ en slikcentrum (Voice and swallowing center) Heerlen the Netherlands

**Keywords:** acoustic analysis, communication, dysarthria, myotonic dystrophy type 1, patient self‐assessment, speech language therapy

## Abstract

**Background:**

Myotonic dystrophy type 1 (DM1) is a heterogeneous neuromuscular disorder characterized by progressive muscle weakness and myotonia. Dysarthria is a known symptom of DM1, but literature is lacking about the patient's own perception in relationship to dysarthria characteristics and severity.

**Aims:**

The aim of the study was to describe the acoustic speech characteristics of dysarthria in patients with DM1, examine the perceptually determined dysarthria severity through speech and language therapy assessment, gather subjective evaluations of speech and intelligibility from patients and relatives and examine the relationship between these outcomes.

**Methods and Procedures:**

The speech of 22 adult patients with DM1 (nine females) was acoustically assessed during spontaneous speech, reading, and maximum performance tasks and analysed using the Praat‐software. Dysarthria severity was rated on a severity scale from 0 (no dysarthria) – 5 (very severe dysarthria/anarthria). Patients and relatives rated the speech with a short questionnaire and a visual analogue scale (VAS).

**Outcomes and Results:**

Acoustic analysis showed a deviant speech rate (SR), articulation rate (AR), maximum phonation volume (MPV), and fundamental frequency range compared to normative values. Perceptually, the dysarthria severity scores varied between 1 (minimal dysarthria) and 4 (severe dysarthria). In more severe dysarthria, SR, AR, and MPV decreased. Patients were sufficiently satisfied about their speech, with no relationship to dysarthria severity. However, the scores of relatives decreased when perceptual dysarthria severity increased.

**Conclusion and Implications:**

As dysarthria severity increased, speech quality and intelligibility declined, particularly when assessed by speech therapists and relatives. Patients with DM1 generally reported minimal conversational restrictions due to dysarthria. Multidimensional measurements may improve the understanding of speech impairment in DM1. Self‐awareness should be a topic in speech therapy interventions.

**WHAT THIS PAPER ADDS:**

*What is already known on this subject*

- Dysarthria is a common symptom in myotonic dystrophy type 1 (DM1). Research has focused on articulatory accuracy and SR. Cognitive decline in patients with DM1 is known to reduce illness insight in approximately half of the cases. However, its impact on self‐awareness of dysarthria, and how this compares to perceptions of relatives and speech therapists, had not been investigated prior to this study. The combination of acoustic and perceptual speech measures with patients’ own perspectives provides new knowledge of speech monitoring in DM1.

*What this study adds to existing knowledge*

- This is the first study to investigate speech in patients with DM1 using both acoustic and perceptual analyses, while simultaneously examining the relationship with patients’ self‐awareness of their speech impairments. The results of this study show a relationship between increasing perceptual dysarthria severity and decreasing acoustic speech performance. Patients often rate their own speech more optimistically than relatives and speech therapists, which suggests a possible role of neuropsychological impairment in reduced self‐awareness of speech deficits in DM1, warranting further research.

*What are the potential or actual clinical implications of this study?*

- The integration of acoustic and perceptual speech assessment allows an objective and accurate diagnosis of dysarthria in DM1. Speech therapy should focus on improving speech clarity and intelligibility by developing exercises for articulatory precision and respiratory control. The discrepancy between how patients perceive their own speech and the opinion of clinicians and relatives suggests that speech therapists may need to focus on raising awareness of the dysarthria and actively involve relatives in the therapeutic process.

## 1 Introduction

Myotonic dystrophy type 1 (DM1) is an inherited progressive neuromuscular disorder characterized by delayed muscle relaxation (myotonia) combined with slowly progressive muscle weakness (dystrophy) (Thornton 2014). DM1 results from an expansion of CTG repeats in the myotonin kinase gene (Harley et al. 1992). DM1 is a multi‐system disorder affecting both muscles and organs, including the central nervous system. Central nervous system symptoms include unawareness of disease burden and difficulties accurately evaluating their own functioning (Baldanzi et al. 2016). These cognitive aspects may indirectly affect communication and self‐assessment of speech,

Many patients with DM1 may present with features of flaccid dysarthria, which results from weakness, fatigue, and hypotonia in the oral and velopharyngeal muscles (De Swart et al. 2004, Hanoun et al. 2022, Maassen et al. 1995, Sjögreen et al. 2007). Tongue myotonia (delayed relaxation of the tongue after contraction) can also be a contributor to the dysarthria (De Swart et al. 2004). Flaccid dysarthria, a motor speech disorder subtype, manifests with observable weakness across one or more components of the motor speech sub‐systems, including articulation, nasal resonance, phonation, respiration, and prosody (Darley et al. 1969, Duffy 2012), which can lead to reduced intelligibility (Hanoun et al. 2022). However, these dysarthric characteristics have been only minimally studied in patients with DM1.

The literature on the various speech subsystems in patients with DM1 is reviewed separately. Firstly, articulation is considered, with research showing that patients with DM1 may experience difficulty producing plosives, because plosives require precise airflow obstruction and release and depend on coordinated bulbar muscle activity (Rietveld and Van Heuven 2016). Muscle weakness, myotonia, and anterior open bite malocclusions may lead to incomplete occlusion of bilabial and labiodental plosives. Patients with congenital and childhood‐onset forms of DM1 compensate for these articulation difficulties by adjusting the place of articulation, using interdental articulation for dental consonants and interdental or labiodental articulation for bilabial consonants (Sjögreen et al. 2018).

Secondly, nasal resonance is addressed. Weakened velopharyngeal muscles may increase nasal airflow during speech in patients with DM1, as suggested by the observed correlation between maximum lip force and nasality (Holmberg et al. 1996, Sjögreen et al. 2018).

Next, phonation and respiration, which are closely interrelated, are discussed. Research shows that respiratory‐phonation weakness leads to a limited duration of maximum sound prolongation and decreased loudness in patients with adult‐onset DM1 (De Swart et al. 2004). Poor laryngeal coordination may further generate turbulent noise or vocal energy during silent intervals (Havner et al. 2023, Mejersjö and Kiliaridis 2017). Additionally, a single case study suggests that dystrophy and myotonia specifically within the laryngeal muscles may result in dysphonia, stridor, and dyspnoea (Ahmadian et al. 2002), but the available evidence is limited and the extent of this occurrence and its impact on voice quality remain unknown.

Finally, temporal aspects of prosody are reviewed, with a focus on SR and pauses. Muscle weakness frequently results in slower SR and frequent, prolonged intra‐sentence pauses (Hong and Byeon 2014), whereas myotonia causes irregularities in speech fluency (Maassen et al. 1995). However, continuous speech may reduce these effects as a result of warming up (De Swart et al. 2007, De Swart et al. 2004). Patients with DM1 show a lower SR than controls during reading, recitation and repetition tasks that require maximal performances (De Swart et al. 2004), while no difference is found in tasks without maximal performances (De Swart et al. 2007). These findings conflict with observations in our clinical setting, where perceptual assessments by speech therapists suggest a faster SR in patients with DM1. It is unclear whether this SR is too fast or if it results from speaking faster than their capabilities due to reduced articulation.

Although dysarthria in DM1 has been studied, uncertainties persist due to limited and outdated literature and diversity in study populations and methodologies. Previous findings primarily rely on maximum performance and repetition tasks, overlooking valuable insights from spontaneous speech and reading tasks. Acoustic analysis incorporating these tasks would greatly contribute to our comprehension of dysarthria in DM1 and potentially establish these speech characteristics as markers for clinical trials (Hanoun et al. 2022).

In addition to the limited literature on speech characteristics in DM1, clinical practice suggests that patients’ self‐assessment of their speech also requires further investigation. In our clinical setting, patients with DM1 often report experiencing little or no speech problems, which contrasts with observations of relatives and speech therapists. The contrast may potentially be explained by the cognitive impairment in DM1, resulting in less accurate self‐evaluation (Baldanzi et al. 2016). However, it is still unclear whether the reduced self‐awareness described in DM1 also affects patients’ ability to assess their own speech or intelligibility. While patients with acquired dysarthria report feeling treated differently and experiencing communication barriers (Walshe and Miller 2011), it remains unclear how much patients with DM1 experience similar limitations.

The study aims to (1) describe the acoustic speech characteristics of dysarthria in adult patients with DM1; (2) examine the perceptually determined severity of dysarthria in patients with DM1 by speech therapists; (3) examine the opinions of patients with DM1 and relatives about the speech and intelligibility; (4) describe the relationships between the perceptual severity of dysarthria, the acoustic speech characteristics, and the opinion of the patients with DM1 and relatives about the speech.

Beyond these specific aims, the study also seeks to contribute to broader goals: enabling a more objective diagnosis of dysarthria, raising awareness about the patients’ self‐assessed severity, and providing new insights into speech monitoring in DM1.

## 2 Method

### 2.1 Patients

Adult patients (age > 18 years) with a genetically proven DM1 diagnosis were recruited between March and May 2024 at the Radboud university medical center, a DM1 expertise centre. Inclusion criteria were Dutch as their native language and presenting with dysarthria during the speech therapy examination. Exclusion criteria encompassed patients unable to read and write and patients in whom any other type of voice, speech, or hearing disorders were raised during the clinical assessment. The included patients were categorised into groups based on the age of the neuromuscular symptom onset and classified as congenital (< 10 years), juvenile (10 years – 20 years), adult‐onset (20 years – 40 years), or late‐onset (> 40 years) DM1 (De Antonio et al. 2016). All patients received an information letter and signed informed consent prior to the examination. The medical ethics committee decided this study did not require a Medical Research Involving Human Subjects Act certification (file 2024‐17072).

### 2.2 Procedure

Experienced speech therapists, each with 5 years – 30 years' experience in neuromuscular disorders, collected recordings, which lasted approximately 10 min, during speech therapy consultations. Each patient completed a set of speech tasks from the Radboud Dysarthria Assessment (RDA) (Knuijt et al. 2017) as part of a larger assessment. The RDA is a standardised set of speech and maximum performance tasks for perceptual speech analysis, supplemented with a qualitative rating form, a severity scale, and a self‐evaluation questionnaire. While the specific speech tasks are presented here, the methods used to perform the measurements are described in the Acoustic Analysis section.

- Response to open‐ended questions about leisure activities, family, and/or work for two minutes.
- These spontaneous speech samples were used to measure the SR and AR.
- Reading task (Dutch reading passage ‘De Koning’)
- Samples were also analysed to assess speech and ARs. Four monosyllabic words with final phoneme /t/ were also selected Table 2 to analyse the accuracy of plosive articulation.
- Sustained production of the phoneme /a: / across the lowest and highest pitches to assess fundamental frequency range. For those unable to glide between pitches, the task was adapted to producing only their lowest and highest pitches. These pitch limits were used to assess the function of the intrinsic laryngeal musculature and its neurological control.
- Production of the sustained vowel /a: / for as long as possible.
- For each patient, a 3 s segment was extracted from the middle of this vowel to analyse voice quality while minimizing distortions at phonation onset and effects of fatigue at the end. Total duration maximum phonation time (MPT) provided insights into breath control and vocal efficiency.
- Produce: ‘Hey Hallo!’ two times as loud as possible. This task was used to assess vocal power and capacity MPV.

The speech tasks were recorded in a room without exterior sounds, by eliminating open windows, doors, or cooling fans. The speech was recorded with the VLC Media Player and a head‐mounted microphone (Sennheiser USB headset CAN ICES‐3(B)/NMB‐3(B)) placed approximately 5 cm from the patient's lips.

Cognition, specifically mental flexibility, was assessed using the subtest ‘verbal fluency’ of the Frontal Assessment Battery (FAB). The FAB is a short neuropsychological test designed to evaluate executive functions related to frontal lobe activity. The verbal fluency subtest was chosen because it measures mental flexibility and shows high sensitivity and specificity for left dorsomedial frontal cortex dysfunction (Van Loon et al. 2007). A cut‐off score of nine was used for cognitive impairment (Van Loon et al. 2007).

### 2.3 Perceptual Assessment

At the end of the consultation, the speech therapist scored the severity of the dysarthria on the 6‐point RDA scale (0 = no dysarthria, 1 = minimal dysarthria, 2 = mild dysarthria, 3 = mild/severe dysarthria, 4 = severe dysarthria, 5 = very severe dysarthria/anarthria) (Knuijt et al. 2017). This score was based on listening to the patients' speech while performing all speech tasks during the consultation. The severity scale of the RDA has proven to have a high interrater reliability.

### 2.4 Patient‐Reported Outcome Measures

Both the patient and his relative were requested to independently rate satisfaction with the patient's speech and intelligibility on a VAS which was presented as a straight line of 10 cm. They were asked to mark the point representing their opinion. Satisfaction with the speech was scored between ‘not satisfied’ and ‘totally satisfied’. Intelligibility was scored between ‘not intelligible at all’ and ‘totally intelligible’. The brief RDA self‐evaluation questionnaire was also administered. This questionnaire has been validated for use in both clinical practice and research as part of the RDA protocol (Knuijt et al. 2017) It consists of seven items in which patients rate the extent of their speech difficulties across different situations, encompassing functional domains (e.g., prolonged and loud speaking), activity and participation domains (speaking to relatives, strangers, in groups, and on the telephone), and social‐emotional impact (perceived degree of impairment). The patients completed the questionnaire by selecting from one of five response options, ranging from 1 (normal) – 5 (most severe impairment).

### 2.5 Acoustic Analysis

The analyses were conducted using the Praat software (Boersma and Weenink 2016) combined with validated automatic scripts (Knuijt et al. 2024, Nijkamp 2018). These scripts are pre‐programmed to calculate specific speech characteristics from a speech recording without the need for manual annotation. The speech characteristics and outcome measures are presented in Table 1 .

**TABLE 1:** The speech characteristics, outcome measures and used speaking tasks.

Speech variable	Outcome measure	Speaking task
HNR	Decibels sound pressure level	Sustained vowel /a: / (2x)
MPV	Decibel	Saying 'Hey Hallo!' as loud as possible
MPT	Seconds	Sustained vowel /a: / (2x)
FFR	Semitones	Gliding on vowel /a:/ up and down (2x)
SR	Syllables/ second	Reading task and spontaneous speech task
AR	Syllables/ second without pauses ≥ 250 ms	Reading task and spontaneous speech task
SI	Seconds	Reading task
PI	Decibel	Reading task

*Note*: HNR: harmonics to noise ratio; FFR: fundamental frequency range; MPV: maximum phonation volume; MPT: maximum phonation time; SR: speech rate; AR: articulation rate; SI: silent interval duration; PI: peak intensity.

**TABLE 2:** Words with voiceless plosive /t/ in the reading passage.

Consonant	Phonetic realisation (IPA)	Orthographic (Dutch)	Orthographic (English)
/t/	[pʏt]	put	well
	[pɑt]	pad	path
	[ʋɑt]	wat	something
	[flœyt]	fluit	flute

Voice quality, which reflects how bright or rough a voice sounds, was assessed using the Harmonics‐to‐Noise Ratio (HNR). HNR evaluates the balance between harmonic sound and noise components (Chandrashekar et al. 2019). Intrinsic laryngeal muscle function and neural control were assessed using Fundamental Frequency Range (FFR). FFR is expressed in semitones as they provide equal musical pitch steps on a logarithmic scale, ensuring consistent reliable cross‐individual comparisons. These measures were compared to normative values from research by Bodt et al. (2015). Vocal power and capacity were evaluated through MPV, which reflects the loudest speech a patient can produce. Breath control and vocal efficiency were measured using MPT, the longest duration a patient can sustain a vowel on a single breath. MPV and MPT were compared to normative values from Knuijt et al. (Knuijt et al. 2019). SR refers to the speed of speaking in general, while AR focuses on the speed of pronouncing syllables, excluding pauses. Pauses are defined as silent intervals of 250 ms or longer (Miller et al. 1984). Audible disfluencies such as repetitions, revisions, and filled pauses like ‘uh’ were also included in the syllable count. The SR and AR of the patients were compared to normative values from Van Borsel and De Maesschalck (Van Borsel and De Maesschalck 2008) and Verhoeven et al. (Verhoeven et al. 2004). The accuracy of plosive articulation was measured using the Silent Interval (SI), which refers to the duration of silence before the explosion, and the Peak Intensity (PI), which is the maximum intensity during the explosion.

### 2.6 Statistical Analysis

Analyses were primarily descriptive and exploratory. Data are presented as means/SD or medians/IQRs as appropriate. Differences are shown visually. Given the small, heterogeneous convenience sample and absence of a priori hypotheses, no significance testing was performed.

## 3 Results

### 3.1 Patient Characteristics

A total of 22 patients (13 men and nine women) with a genetically confirmed diagnosis of DM1 participated in this study after the exclusion of one patient due to the absence of dysarthria (perceptual severity score of 0) and one patient due to insufficient reading ability. Patient characteristics are shown in Table 3. As illustrated in Table 3, the distribution of the FAB scores and the DM1 types were too skewed to allow for further analyses.

**TABLE 3:** Characteristics of all DM1 patients.

	N	M	SD	Range
Sex (M/F)	13/9			
Age (y)		51	15.17	23 ‐ 85
Age of disease onset (y)		37	15.29	12 ‐ 78
DM1 type		—	—	—
Juvenile	3			
Adult‐onset	13			
Late onset	6			
Perceptual severity score				
1	7			
2	7			
3	6			
4	2			
FAB (Subtest: verbal fluency)		14.59	6.05	5 ‐ 32
Score ≤ 9	4			
Score > 9	18			

*Note*: y: years; N: number of observations; M: mean; SD; standard deviation; FAB: Frontal Assessment Battery.

### 3.2 Perceptual Severity Score

Perceptual speech analysis revealed a median of 2 on the dysarthria severity score (IQR = 2, range: 1 – 4). The patients presented with minimal (*n* = 7), mild (*n* = 7), mild/severe (*n* = 6), and severe (*n* = 2) dysarthria severity.

### 3.3 Self‐reported Speech Perception

#### 3.3.1 VAS Score

All 22 patients completed the satisfaction with speech and intelligibility VAS scales. However, six scores from relatives were missing due to the absence of a family member or partner during the assessment Table 4. 28% of the patients reported high satisfaction with their own speech (mean score of 9.08), and 23% found their speech highly intelligible (mean score of 8.80). 82% of the patients gave similar or nearly identical scores (within 1.5 points) for both scales, indicating comparable scores for speech and intelligibility.

**TABLE 4:** VAS scores of patients and relatives.

	VAS1P	VAS2P	VAS1R	VAS2R
N	22	22	16	16
Mean (range)	6.57 (2.5 – 10.0)	6.00 (3.0 – 9.5)	6.66 (3.0 – 9.0)	6.03 (3.0 – 9.5)
SD	2.16	2.08	1.75	1.87
Scores N (%)				
> 2,5 ≤ 5	8 (36%)	9 (41%)	3 (19%)	7 (44%)
> 5 ≤ 7.5	8 (36%)	8 (36%)	8 (50%)	5 (31%)
> 7.5	6 (28%)	5 (23%)	5 (31%)	4 (25%)

*Note*: VAS1P: patients score of VAS question 1 (satisfaction with speech quality); VAS2P: patients score of VAS question 2 (intelligibility); VAS1R: relatives score of VAS question 1 (satisfaction with speech quality); VAS2R: relatives score of VAS question 2 (intelligibility); N: sample size; SD: standard deviation.

#### 3.3.2 RDA Questionnaire Score

All patients completed the RDA self‐evaluation questionnaire. Median scores for the seven questionnaire items ranged from 1 – 2, with interquartile ranges of 1 and 2. Four patients (18%) gave the minimum score of one for all questions, and seven patients (32%) gave a score of either 1 or 2, consistent with the absence of speech issues in social contexts, or occasional difficulty when fatigued (score of two). Two patients gave the maximum score twice. One felt excluded during group discussions and experienced dysarthria as the most severe limitation of DM1, while the other patient could not speak with a raised voice or for prolonged periods. The two highest severity scores were not observed among patients with severe dysarthria, but among patients with mild/severe dysarthria. This suggests a lack of apparent association between dysarthria severity and these scores.

### 3.4 Acoustic Speech Variables

**FIGURE 1:**
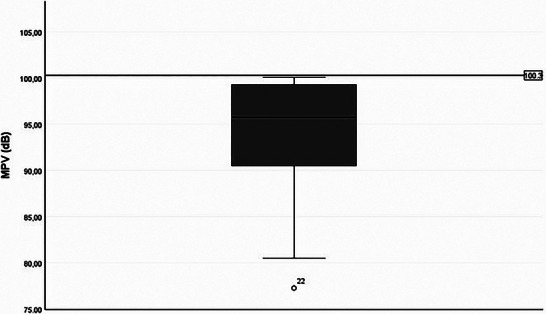
Boxplot of MPV in patients with DM1 with reference value line.

**FIGURE 2:**
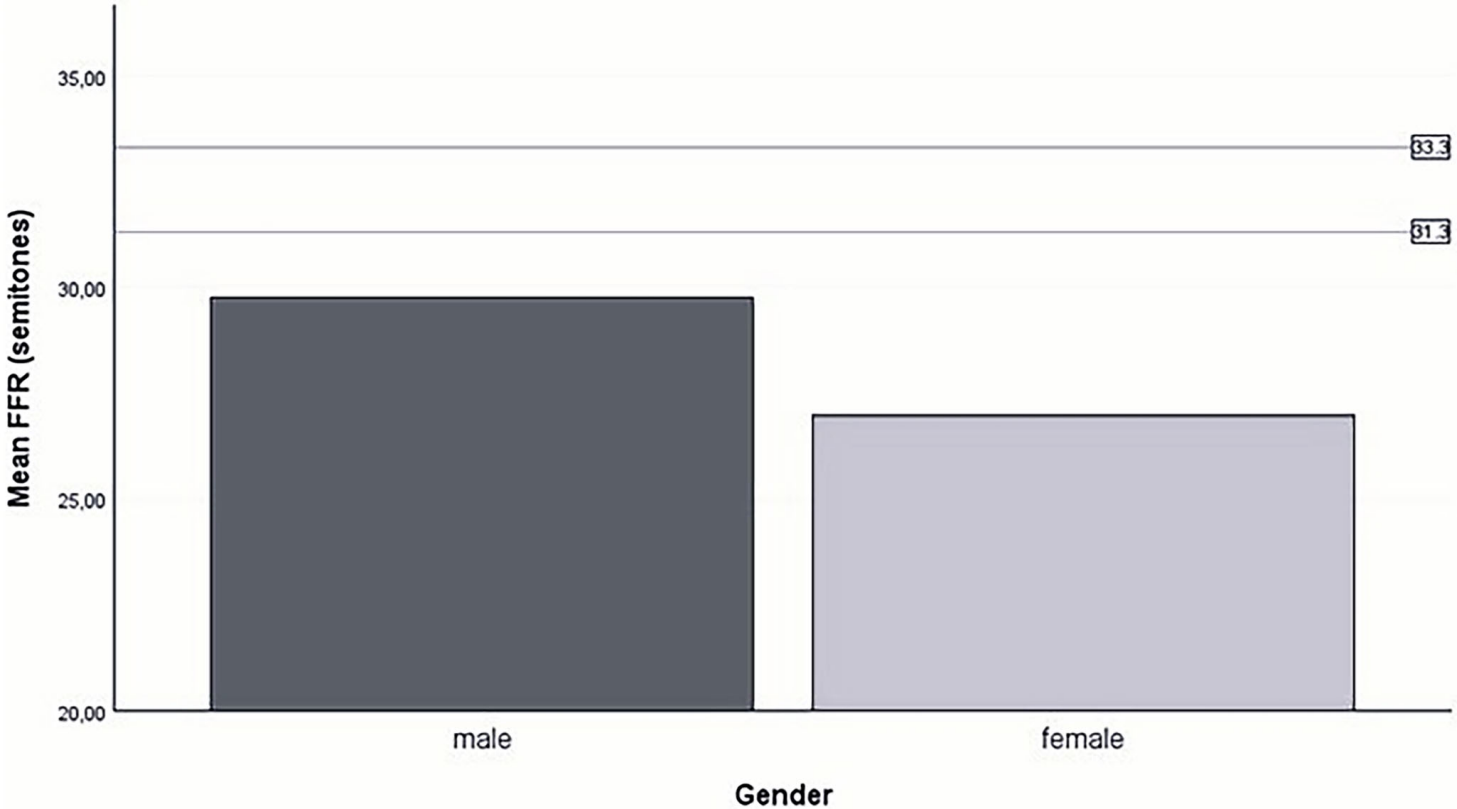
Bar chart of mean FFR by sex with reference value lines of male (dark gray) and female (light gray) speakers.

**FIGURE 3:**
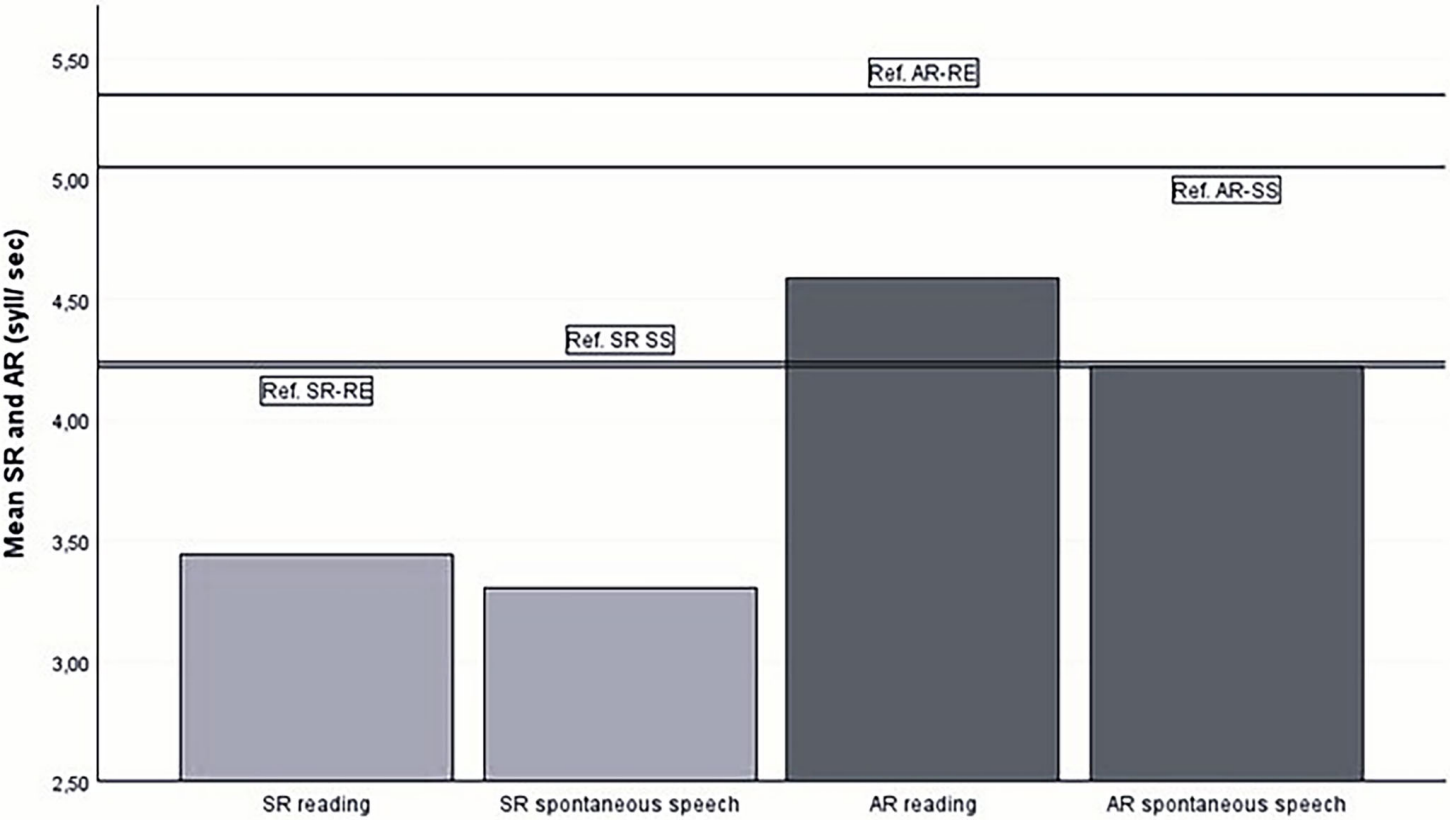
Bar chart of mean SR (light gray) and AR (dark gray) in patients with DM1 with reference value lines.

All descriptive statistics of the patient's speech characteristics are presented in Appendix 1. In Tables 5 and 6, the patient's data and the reference values are depicted. Patients with DM1 showed a lower MPV Figure 1, and both men and women showed a smaller FFR Figure 2. Additionally, patients with DM1 showed a slower SR during spontaneous speech and reading. They also had a slower AR during reading and spontaneous speech Figure 3. The MPT of patients with DM1 is shorter than the reference value.

**TABLE 5:** Mean values of the acoustic variables in patients with DM1 and the mean reference values of non‐pathological speakers.

	DM1		Ref‐value	
	M	SD	M	SD
HNR (dBSPL)	19.37	4.46	17.49	NA
FFR male	29.76	5.75	33.30	5.88
FFR female	26.97	5.92	31.30	9.37
SR‐SS (syl/s)	3.31	0.52	4.24	NA
SR‐RE (syl/s)	3.44	0.54	4.22	NA
AR‐SS (syl/s)	4.22	0.48	5.05	NA
AR‐RE (syl/s)	4.59	0.39	5.35	NA

*Note*: HNR: harmonics to noise ratio; FFR: fundamental frequency range; SR: speech rate; AR: articulation rate; SS: spontaneous speech; RE: reading; dB: decibel; s: second; syl: syllable; M: mean; SD: standard deviation; NA: not available; Ref‐value: reference value.

**TABLE 6:** Medians of the acoustic variables in patients with DM1 and median reference values of non‐pathological speakers.

	DM1		Ref‐value	
	Mdn	IQR	Mdn	IQR
MPV (dB)	95.80	8.90	100.30	1.70
MPT (s)	13.51	11.26	19.40	10.20

*Note*: MPV: maximum phonation volume; MPT: maximum phonation time; dB: decibel; s: second; Mdn: median; IQR: interquartile range; Ref‐value: reference value.

Most patients with DM1 pronounced the plosive /t/ with a very short or absent SI (range between 0 s and 0.112 s) Appendix 1. This was difficult to examine due to very small explosions, air leakage, or the production of multiple explosions during the articulation. Patients showed a mean PI of 32.03 dB varying between 48.02 dB and 86.26 dB. Additionally, a, long explosion duration was noticeable in some patients, making the pronunciation of the /t/ sounded more like a /s/.

### 3.5 Effect of Dysarthria Severity

**FIGURE 4:**
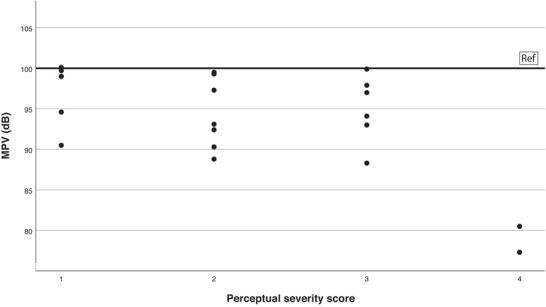
Scatter plot of MPV in patients with DM1 by perceptual severity score.

**FIGURE 5:**
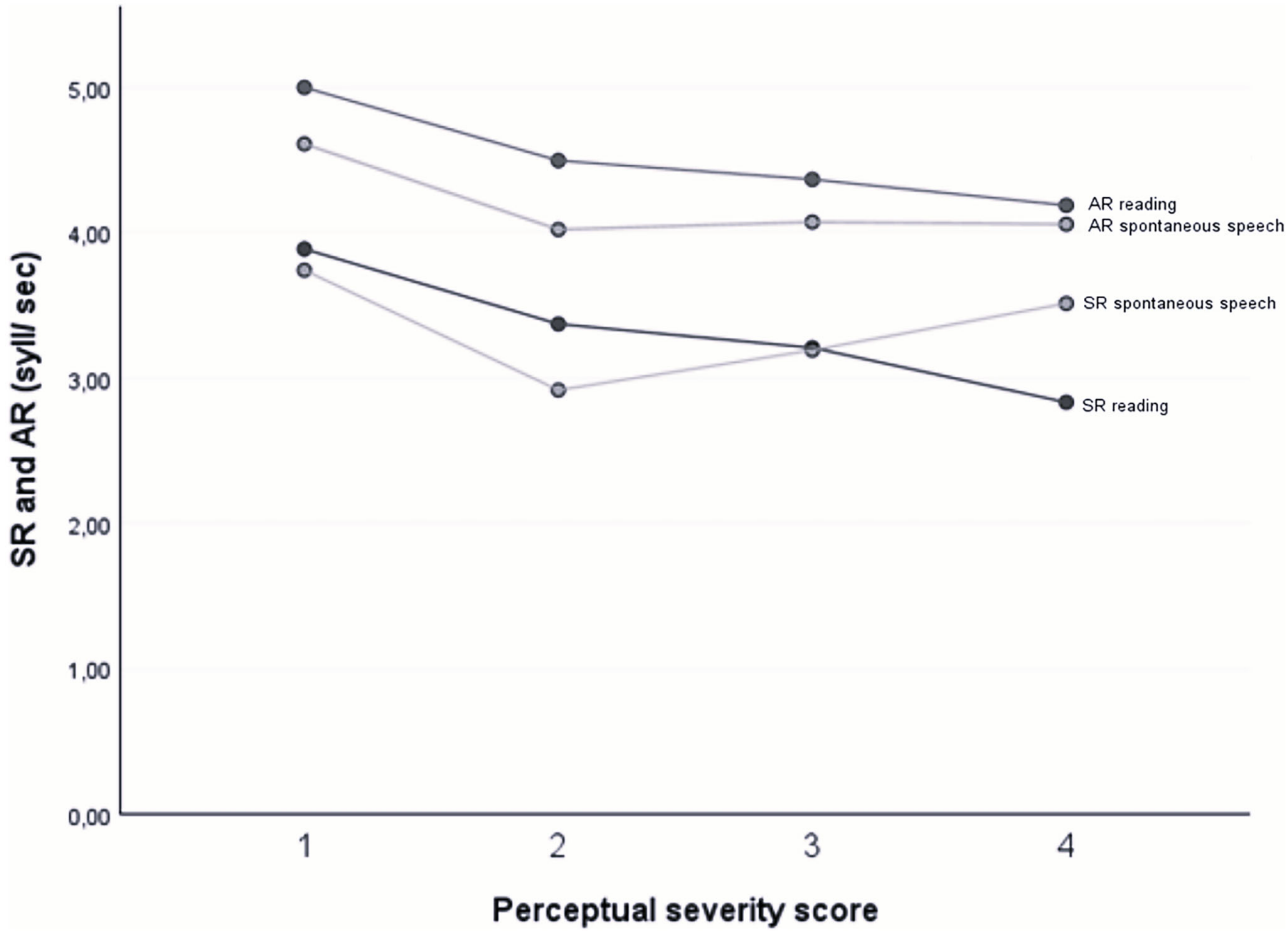
Line charts of mean SR and AR in patients with DM1 during reading in relation to dysarthria severity.

In Figures 4 and 5 we plotted the MPV and SR against the perceptual severity scores.

**FIGURE 6:**
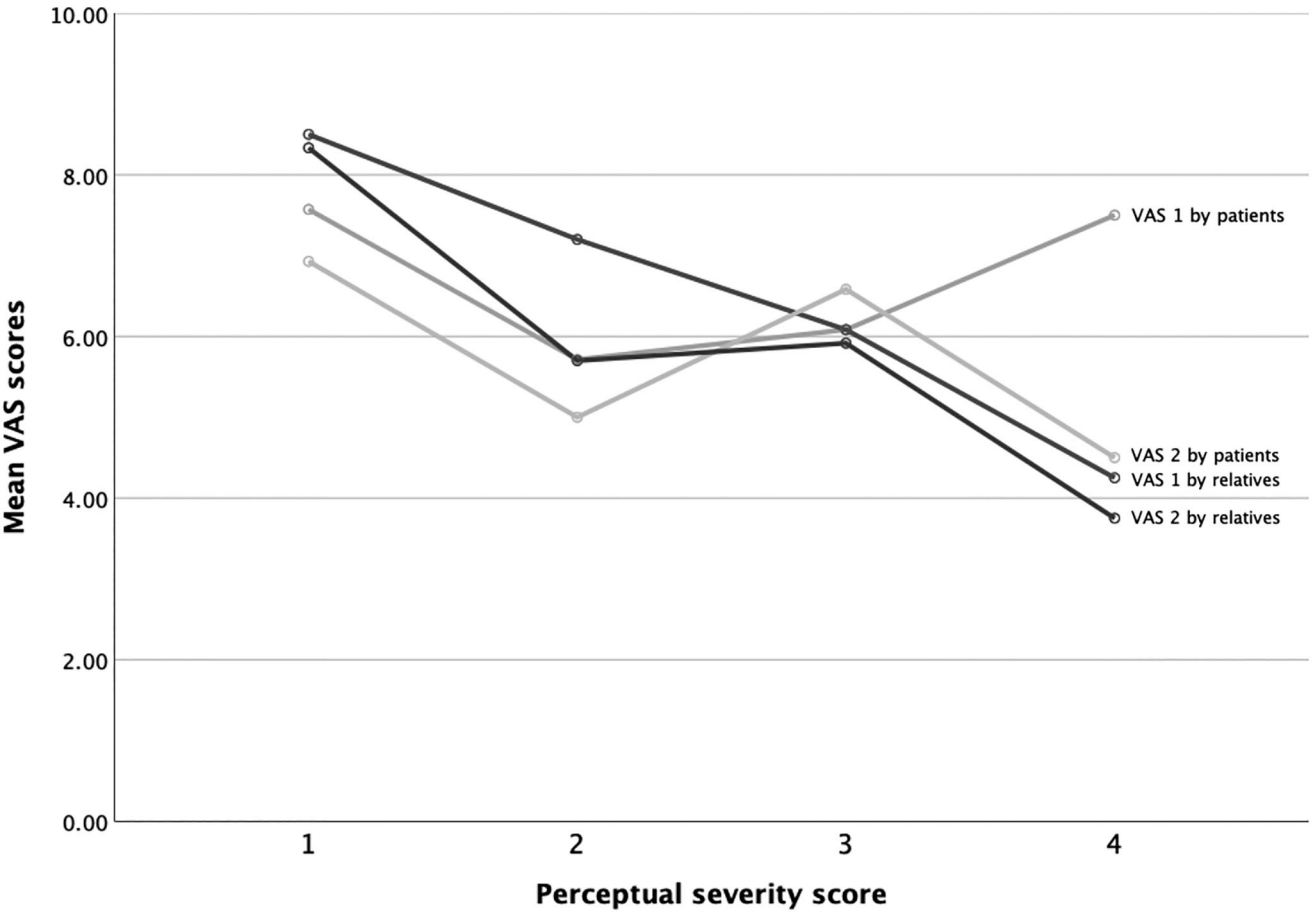
Line chart of mean VAS scores of patients and relatives related to dysarthria severity.

The decreased MPV is mainly determined by the patients with severity score of four, which consists of only two patients. So, this finding appears less compelling considering the limited number of patients with severe dysarthria in the study. As the perceptual severity score increased, AR and SR in the reading condition decreased Figure 5. The VAS scores of the relatives decrease (less satisfaction) when dysarthria severity increases Figure 6. This pattern is not visible in the VAS scores of the patients.

## 4 Discussion

In this study, we acoustically analysed the speech characteristics of dysarthria in patients with DM1, perceptually assessed dysarthria severity through speech and language therapy clinical assessment, gathered subjective evaluations from patients and their relatives, and examined relationships between these outcomes. Given the exploratory design and limited sample size, we focused on descriptive patterns rather than statistical significance.

The patient group demonstrated sufficient variability in dysarthria severity, with an even distribution across minimal, mild, and mild/severe cases, but few severe cases. This distribution indicates that the patient cohort was well‐suited for an initial investigation into the acoustic properties of dysarthric speech in DM1, yielding valuable preliminary insights. According to the word fluency test of the FAB, no patient exhibited cognitive impairment, however their VAS scores for speech varied widely. Given that self‐assessment is affected in other functional domains in DM1 (Baldanzi et al. 2016), it seems plausible that speech‐related self‐awareness may follow a similar pattern. However, cognitive deficits were assessed only in terms of mental flexibility using the verbal fluency test, which represents just a small aspect of overall cognition, due to time constraints during clinical consultations. A comprehensive assessment across multiple cognitive domains is therefore required to draw reliable conclusions about overall cognitive functioning.

### 4.1 Acoustic Speech Characteristics of Patients With DM1

Patients with DM1 exhibited a lower MPV and a reduced fundamental frequency range (FRR). The lower MPV is consistent with previous literature associating reduced maximum performance tasks with respiratory‐phonatory weakness (De Swart et al. 2004). However, the current study does not confirm the prediction of an abnormal voice quality as a consequence of this weakness (Ahmadian et al. 2002, De Swart et al. 2004, Rampello et al. 2016). In Duchenne muscular dystrophy (DMD), respiratory muscle weakness interferes with the maintenance of subglottic pressure required for voice production (Hijikata et al. 2022). This raises the possibility that respiratory muscle weakness may similarly impact voice production in DM1. However, the contrasting results in this study may be attributed to the predominance of mild‐to‐moderate dysarthria cases and potentially unaffected pulmonary capacity in the included patients. Since lung impairment in DM1 typically emerges at later disease stages due to its slow progression, early symptoms may remain undetectable (Akpa et al. 2024, Thil et al. 2017).

Patients with DM1 showed a slower SR and AR in both reading and spontaneous speech, a pattern consistent with flaccid dysarthria patterns (Hong and Byeon 2014). However, in contrast, De Swart et al. (2007) found no significant difference in SR between patients with DM1 and controls. This discrepancy could be explained by methodological factors or the influence of myotonia, which causes irregularities in speech fluency and unpredictable speech (Maassen et al. 1995). The effects of myotonia tend to diminish with continuous speech production, as a result of a warming‐up effect (De Swart et al. 2007, De Swart et al. 2004). The reading task in the present study may have been too brief to induce this effect, thereby contributing to the observed slower overall SR. Additionally, the perceptual impression of an abnormally fast SR may stem from reduced consonant articulatory precision, causing listeners to misinterpret faster‐than‐expected speech production (Koreman 2006). Further research is needed to verify this hypothesis.

### 4.2 Effect of Dysarthria Severity

As the dysarthria severity increased, the MPV of patients with DM1 decreased.

Similar patterns have been observed in Parkinson's disease (PD), Amyotrophic Lateral Sclerosis (ALS), and Duchenne muscular dystrophy (DMD), where speech impairment has been linked to respiratory dysfunction (Di Pietro et al. 2022, Hijikata et al. 2022, Sarmet et al. 2024). Patients with DM1 and a more severe dysarthria showed slower SR and AR during reading. This supports the hypothesis that dysarthria severity represents a critical predictor for deviations in prosody and articulation (Joshy et al. June 2023). However, the presence of this correlation in other prosodic DM1 features, such as intonation, remains unclear. Relatives of patients with more severe dysarthria reported greater difficulty in comprehending speech and higher levels of dissatisfaction regarding speech intelligibility. This pattern was not reflected in the patients’ own VAS scores, which might suggest that dysarthria severity is not so evident to patients themselves. However, given the small sample of relatives, missing data, and high variability, these findings should be interpreted with caution and warrant further investigation.

### 4.3 Limitations and Recommendations

Due to missing data and high variability in the scores of relatives, no reliable comparison can be made between the VAS scores of patients and relatives. Additionally, analysing the influence of cognitive impairment and other, for example psychological, factors on speech production and self‐awareness was not possible, as most included patients exhibited minimal cognitive deterioration, according to the FAB scores. Collaboration with neuropsychologists is recommended in future research to disentangle cognitive from emotional and motivational influences on speech self‐awareness.

The inclusion of both adult‐ and juvenile‐onset patients increased heterogeneity, which may have obscured subtype differences. Future research should stratify analyses by disease onset and severity. Inclusion of sufficient numbers of patients is needed, including data of disease severity or genetic data such as CTG repeat length. The relationship between cognition and self‐awareness of dysarthria as well as the relationship between patients' self‐assessment and relatives’ evaluations warrants further research. Follow‐up studies could examine whether cognitive or psychological factors such as disease insight and emotional coping mechanisms play a role in these relationships.

During the spontaneous speech analysis, the speech therapist's speech (e.g., questions or responses) interfered with the acoustic analysis, as the script misclassified this input as patient syllables. Smaller segments without interruptions were selected to address this, though this method reduced the amount of speech material. To prevent this issue in future research, the guidelines for spontaneous speech tasks should be more specific, clearly stating that the patient narrates a dialogue for two minutes without turn‐taking. Additionally, automatic syllable annotations should be thoroughly reviewed and manually adjusted if necessary.

It cannot be determined whether the SI and PI in patients with DM1 deviate from non‐pathological speakers, due to the absence of reference values in the literature. Additionally, the silent interval duration is an unsuitable acoustic measure for dysarthric speech, due to turbulence noise and voicing energy found in the spectrogram, which was also found in the articulation of other dysarthric speakers (Kent et al. 1999).

The normative values used for the other acoustic speech variables were also problematic, as they did not consistently correspond to the speaking task or individual patient characteristic, and were sometime only available by sex. Including a control group of non‐pathological speakers matched by sex and age and testing a larger group of patients with DM1 would improve the reliability of the acoustic analysis in follow‐up studies. Longitudinal studies may help to better understand the progression of dysarthria in DM1 and identify critical stages for preventive or therapeutic intervention.

### 4.4 Clinical Implications

The integration of acoustic and perceptual speech assessments can enhance the objectivity and accuracy of dysarthria diagnosis in patients with DM1. Moreover, Praat software, in combination with validated automatic scripts, holds potential for clinical application, allowing for the objective monitoring of disease progression in DM1.

Our findings indicate that reduced MPV and slower AR are particularly linked with perceptual dysarthria severity. Speech therapy could therefore focus on exercises that combine respiratory control and articulatory precision, while also addressing patients’ self‐awareness and involving relatives in feedback moments. Involving relatives in therapy could enhance insight and communication strategies.

Although evidence is limited, the observed difference between patients’ self‐assessments and their relatives' perceptions suggests that involving relatives in the therapeutic process of patients with DM1 may be valuable, a recommendation that warrants confirmation in future research. This approach may foster a more accurate understanding of the communicative challenges faced by patients with DM1, ultimately improving both speech therapy outcomes and interpersonal communication.

## 5 Conclusion

In patients with DM1, dysarthria was evident in both acoustic and perceptual measures. Greater perceptual severity was associated with larger deviations in certain acoustic features.

Patients with DM1 generally reported minimal conversational restrictions due to dysarthria. However, as dysarthria severity increased, the relatives reported more problems regarding speech quality and intelligibility.

Future research with a larger sample size and a control group is recommended to further validate and expand upon these findings. This study provides new insights into the speech characteristics of dysarthria in DM1, as well as patients’ awareness of their speech and intelligibility. The findings contribute to a better understanding of dysarthria in DM1 among healthcare professionals, which may support more effective referral policies and tailored intervention strategies.

## Funding

The authors have nothing to report

## Ethics Approval Statement

The medical ethics committee of the Radboud university medical center decided this study did not require a Medical Research Involving Human Subjects Act certification (file 2024‐17072).

## Patient Consent Statement

All patients received an information letter and signed written informed consent prior to the examination.

## Conflicts of Interest

The authors declare no conflicts of interest.

## Permission to Reproduce Material From Other Sources

No material from other sources has been used in this manuscript. All figures, tables, and text are original and created by the authors.

## Data Availability

The data that support the findings of this study are not publicly available due to the privacy of research patients but are available from the corresponding author [SK].

## Summary of Statistic Characteristics of Acoustic Variables in Patients With DM1

**Table:**

	M	SD	SEM	Mdn	IQR	Min–Max	Var
HNR (dBSPL)	19.37	4.46	0.95	—	—	10.58 – 28.37	19.89
FFR (semitones)	28.62	5.85	1.25	—	—	15.16 – 37.96	34.18
MPV (dB)	—	—	—	95.80	8.90	77.30 – 100.10	39.80
MPT (s)	—	—	—	13.51	11.26	7.00 – 35.00	60.07
SR‐SS (syl/s)	3.31	0.52	0.11	—	—	2.42 – 4.38	0.27
SR‐RE (syl/s)	3.44	0.54	0.11	—	—	2.34 – 4.10	0.29
AR‐SS (syl/s)	4.22	0.48	0.10	—	—	3.55 – 5.34	0.23
AR‐RE (syl/s)	4.59	0.39	0.08	—	—	3.89 – 5.29	0.15
SI [pʏt] (s)	0.07	0.02	—	—	—	0.04 – 0.11	0.00
SI [pɑt] (s)	0.06	0.03	—	—	—	0.01 – 0.10	0.00
SI [ʋɑt] (s)	0.04	0.03	—	—	—	0.00 – 0.10	0.00
SI [flœyt] (s)	0.04	0.02	—	—	—	0.00 – 0.07	0.00
PI [pʏt] (dB)	60.05	5.69	—	—	—	48.02 – 82.53	33.95
PI [pɑt] (dB)	61.46	8.62	—	—	—	48.05 – 86.26	77.82
PI [ʋɑt] (dB)	64.83	8.71	—	—	—	51.02 – 82.32	79.48
PI [flœyt] (dB)	61.79	8.76	—	—	—	48.02 – 82.53	80.39

*Note*: HNR: harmonics to noise ratio; FFR: fundamental frequency range; MPV: maximum phonation volume; MPT: maximum phonation time; SR: speech rate; AR: articulation rate; SS: spontaneous speech; RE: reading; SI: silent interval duration; PI: peak intensity; dB: decibel; s: second; syl: syllables; M: mean; SD: standard deviation; SEM: standard error of mean; Mdn: median; IQR: interquartile range; Var: variance.
